# Unsung heroes in health education and promotion: How Community Health Workers contribute to hypertension management

**DOI:** 10.3389/fpubh.2023.1088236

**Published:** 2023-02-23

**Authors:** Kim Bush, Carlea Patrick, Kimberly Elliott, Michael Morris, Yordanos Tiruneh, Paul McGaha

**Affiliations:** ^1^Department of Preventive Medicine and Population Health, University of Texas at Tyler Health Science Center, Tyler, TX, United States; ^2^Department of Health Policy, Economics, and Management, University of Texas at Tyler Health Science Center, Tyler, TX, United States; ^3^University of Texas Southwestern Medical Center, Dallas, TX, United States

**Keywords:** Community Health Workers, health outcomes, health education, health promotion, self-management

## Abstract

Rural communities are noted as having poor health outcomes. Rural areas experience barriers to care primarily due to a lack of resources, including education, health insurance, transportation, and social support. Additionally, poor health outcomes are a consequence of poor health literacy skills. Community Health Workers (CHWs) are utilized as a resource to combat these issues. This study focused on a CHW led Self-Management Blood Pressure (SMBP) program offered through the University of Texas at Tyler Health Science Center. The goal of the program was to improve management of hypertension through awareness, education, navigation, advocacy, and resource assistance. The SMBP program included structured workshops and regular follow-up with participants including connections to community resources and social support. CHWs worked closely with physicians providing bi-directional feedback on referrals and engagement of communities through outreach events. Furthermore, CHWs aided to bridge cultural or linguistic gaps between service providers and community members. Data is provided indicating this CHW-led intervention played a significant role in improving hypertension through education of how to make lifestyle changes that impact overall health and quality of life. Participants gained knowledge encouraging them to create lifelong healthy habits, coping skills, stress management, self-care, and accountability. Through this innovative approach, participants thrived in the supportive and encouraging environment led by CHWs as well as improved their blood pressure management.

## Introduction

Cardiovascular disease and stroke are respectively the number one and five leading causes of death in the United States (1) and in Northeast Texas (2). Northeast Texas is documented as one of the least healthy regions of the state, with a prevalence of chronic disease significantly above both state and national averages (2). While these statistics have remained stable over time (1, 2), it is widely recognized that hypertension is a modifiable risk factor for both deadly conditions (3). Improving the control of hypertension is a complex task, which is compounded in rural regions such as Northeast Texas. It is well documented that in rural communities there is less access to care, the quality of care is often lower, and community resources are less readily available to support health within the community (4, 5). A class of strategies that has been utilized to attempt to overcome the complexity of hypertension management among rural (6, 7) and other underserved populations (8) are Community Health Workers (CHW) led interventions.

CHWs are known to build community capacity in efforts to improve health outcomes. Additionally, CHWs play a significant role in shifting the trajectory of chronic disease by encouraging lifestyle changes that impact overall health and quality of life (9). CHWs also serve to bridge cultural and/or linguistic gaps between service providers and community members. By providing education and resources to improve health literacy about symptoms, negative consequences, and treatment outcomes associated with chronic illness, CHWs empower individuals to play an active role in reducing the severity of their chronic illness.

CHWs play an important role in their communities, providing services at the individual and group level (10, 11). According to the American Public Health Association (12), CHWs are trusted in communities they serve which results in improved relationships between community members and health and social service organizations. Additionally, given proper training, CHWs provide health knowledge through individual and community capacity building through various screening and educational activities (13). Furthermore, Hartzler et al. (14) observed that CHWs provide self-management support to patients through counseling involving collaborative goal setting, problem-solving, and action-planning. Their strength derives in part from their building relationships on trust, emotional attendance, and authenticity (15). Although CHWs are widely known across the nation, the profession may not be utilized to its potential or fully recognized as essential in the broader landscape of chronic disease management.

CHW led interventions have been shown to be effective in the management of non-communicable health conditions (16). For example, the Education to Promote Improved Cancer Outcomes (ÉPICO) project, utilized Spanish-speaking CHWs or promotors, to enhance cancer knowledge among residents of the Rio Grande Valley of Texas (17). Similarly, CHW led interventions have been effective in changing health behaviors among minority populations with diabetes (18). The evidence on CHW's influence on the prevention and management of hypertension extends at least back to the 1970s, with benefits demonstrated in improved health related knowledge and behaviors, and blood pressure reduction (19). More recent systematic reviews by Kim et al. (20) Scott et al. (21), and Cabellero et al. (22) further illustrate that CHW led interventions can be effective in improving the management of chronic disease, including hypertension, among rural and other vulnerable populations.

This study examines the outcomes of a hypertension focused CHW-led Self-Management Blood Pressure (SMBP) program operated by the University of Texas at Tyler Health Science Center. The population of interest is people living with hypertension in Northeast Texas. This study: (1) describes a CHW-led SMBP workshop series designed to improve health outcomes among patients with hypertension receiving care at a rural academic health center; (2) evaluates the program's impact on participant knowledge and behavior regarding blood pressure management before and after completion of the program; (3) assesses the effect of the intervention on hypertension control from blood pressure measures taken at baseline and at the end of the program; and (4) reports participants perceptions and experiences of the program.

## Materials and methods

The University of Texas at Tyler Health Science Center's Self-Management Blood Pressure (SMBP) program is a multi-component lifestyle change intervention that combines and adapts evidence-based components from lifestyle change and hypertension management curricula disseminated by the National Heart, Lung, and Blood Institute (23) and the American Heart Association (24) and TARGET:BP collaboration between AHA and American Medical Association (25). Using these materials, a hybrid curriculum was developed into a 12-week hypertension-management workshop series. Additional materials were provided by the Texas Department of State Health Services (DSHS).

The process began with an orientation session during which participants learned more about the program and completed all required paperwork including an informed consent form before participating in the program. Participants who agreed to join the program were provided blood pressure monitors to use throughout the program and upon graduation they were allowed to keep the apparatus. Participants were taught how to use the blood pressure monitor according to TARGET:BP (26) guidelines and tasked with checking their blood pressure twice daily (once in the morning and once in the evening). Participants were also required to record their daily BP (blood pressure) readings in a blood pressure booklet (My Blood Pressure Passport) Texas Department of State Health Services and Texas Health and Human Services (27) developed by the Texas Heart Disease and Stroke Program and Health Promotion and Chronic Disease Prevention section through the Texas Department of State Health Services (DSHS) and Texas Health and Human Services (HHSC). Additionally, participants were encouraged to share this document with their primary care providers. The workshop participants met bi-weekly over 12 weeks for ~1 h. During these sessions, CHWs provided heart health education to the participants and collected blood pressure readings from the preceding 2-week period. These data were de-identified and stored electronically in a secured file. De-identified data was also shared with DSHS upon cohort graduation. Participants received a $5 gas/gift card upon attendance and participation in the bi-weekly sessions. Participants who completed the entire workshop series were awarded the blood pressure monitor that they used during the program as well as a certificate of successful completion upon graduation. Furthermore, participants were invited to attend a 3-, 6-, 9-, and 12-month follow-up reunion where they acquired additional educational information as well as continued support for maintaining their blood pressure at healthy levels. As an additional incentive, participants were also presented with a $5 gas/gift card for attending post-program follow-up sessions.

The SMBP program is a community-based health program therefore participants self-selected into the program. No statistically based sampling method was employed in the selection of participants, it was a convenient sample. Participants in the SMBP program were recruited from the East Texas community at large through several methods: (i) The primary modes of recruitment were through referrals from University of Texas Health East Texas (UTHET) physician clinics and by word-of-mouth referrals from persons who had previously participated in the program. (ii) UTHET Emergency Medical Services Mobile Integrated Health unit also provided referrals for some of their patients to the SMBP program. (iii) Community outreach fairs and presentations at assisted living centers and the Tyler Library contributed the remaining SMBP participants.

The SMBP sessions included in this study were conducted from August 2019 to June 2022. Three separate data sets, capturing different dimensions of program performance, were collected from persons who enrolled in and participated in the SMBP program. These were (1) the assessment of changes in participant knowledge and behaviors, (2) change in systolic and diastolic blood pressure and, (3) the participants' self-reported experiences of completing the SMBP program.

### Assessment of changes in participant knowledge and behaviors

Participants completed knowledge and skills assessments related to blood pressure management at the start of the program and then upon program completion. The pre-test was comprised of a 5-question knowledge exam followed by a three-question self-assessment of the participant's skills for controlling hypertension. This pre-test was self-administered at the time the individual enrolled in the program. The post-test repeats the questions from the pre-test and is administered at the graduation ceremony from the program. Because results from the pre- and post-test were collected anonymously and tabulated in the aggregate for record retention, we were unable to present measures of significance for the variation in the data.

### Assessment of changes in systolic and diastolic blood pressure

CHWs taught participants how to accurately take their own blood pressure using the automated arm-cuff monitor so that they were able to record daily blood pressure readings in their booklet, My Blood Pressure Passport.

A one group pre-test/post-test design was utilized to assess changes in the participants' mean blood pressure, with separate analyses conducted for changes in systolic and diastolic blood pressure. The pre-test time point was defined as the time of the blood pressure reading taken during the program's first-week session. The post-test time was defined as the time of the measurement conducted during the program's last session week 12. Participants measured their blood pressures in millimeters of mercury using the Omron 7 Series Blood Pressure Monitor and following American Heart Association/American Medical Association TARGET:BP: SMBP guidelines (26). Separate paired sample *t*-tests were utilized to assess the hypotheses that there were no differences between pre- and posttest mean systolic and diastolic blood pressures for the study population. Within group changes in mean blood pressure were assessed for each participant's characteristics available in the data, including age, gender, race, ethnicity, education level, body mass index (BMI), whether the individual was currently on antihypertensive medications, whether they had ever smoked, and whether they were subject to additional comorbidities, diabetes and/or cardiovascular disease. Based on the demographic composition race was defined in this study as being White or Black. Ethnicity was defined as either Hispanic or non-Hispanic. Racial and Ethnic categories are not considered to be mutually exclusive, for example, an individual can be Black and Hispanic or Black and non-Hispanic. Race and Ethnicity data were self-reported by participants. Underlying assumptions for the use of paired *t*-tests were assessed, with no influential outliers noted, and the results of a Shapiro-Wilk test of normality met the requirement for approximate normality. All analyses were conducted using STATA version 16. Statistical significance was set at the 0.05 level.

### Assessment of participant experience

The final data source utilized in this study was a participant experience survey completed at the end of the week 12 intervention. This was a self-administered survey. Responses were anonymous and tabulated at the aggregate level for record retention. The instrument was comprised of five-questions that used a Likert format gauging agreement with the base question on a scale of strongly agree to strongly disagree. All individuals who finished the SMBP program completed at least part of the survey. Because response data were retained at the aggregate level, we were not able to calculate measures of significance for variation in responses. In addition to the formatted questions, participants were invited to provide any additional comments in free form text. For the purposes of reporting here, the responses were divided into four categories, (i) including constructive comments about the CHWs who ran the workshop, (ii) the workshop content, (iii) delivery mode, and (iv) the overall experience with the program.

## Results

Total enrollment for these SMBP cohorts was 242. Enrollment in the program consists of a participant's agreement confirmed by a signed consent to participate in the program. The consent was delivered to potential participants by email and then electronically signed and returned prior to attending the orientation session. The actual number of participants who started the program was 212, which includes a signed consent form, attendance at the orientation or session one, and providing a minimum of one blood pressure reading. Of these enrollees, 197 completed the program by regularly providing blood pressure readings. However, five of the 197 participants failed to complete all 12 weeks of the program and thus did not provide blood pressure readings at the final week of the program leaving us with 192 subjects included in the blood pressure analytical file. The decline from participant enrollment (242) to participants who completed the program (197) could be attributed to multiple factors including, lost to follow-up, family emergencies, COVID-19 pandemic related stress and/or anxiety, over commitment, and other causes. Comparison of the individuals who were dropped from the study sample with those included in the analysis showed no statistically significant differences in gender (*p* = 0.32), race (*p* = 0.43), age (*p* = 0.65), or education level (*p* = 0.28).

Table 1 shows the demographic and health-related characteristics of participants in the program. Our sample skewed older (69.4%) and over three fourth (76.6%) were female. Whites (72.9%) were the predominant racial group (Blacks 27.1%) represented in the study population and non-Hispanics (82.3%) were the more prevalent ethnic group (Hispanics 34.0%). The educational level of participants was evenly distributed, with a slight majority having earned at least some college credit. BMI skewed significantly toward obesity, with 65.1% of participants falling into this category. Similarly, participants were highly likely to be currently on blood pressure medication (73.9%). Regarding comorbidities, 31.4% reported being diabetic, while 24.7% suffered from diagnosed cardiovascular disease. Fewer than a quarter (22.4%) of the participants reported a history of smoking.

**Table 1:** Characteristics of study participants.

**Characteristic**	**Study population**
	***N*** **(%)**
**Overall**	192
**Age in years**	
20–39	17 (8.85)
40–59	61 (31.77)
60–79	106 (55.21)
>79	8 (4.17)
**Sex**	
Female	147 (76.56)
Male	45 (23.44)
**Race** ^#^	
Black	52 (27.08)
White	140 (72.92)
**Ethnicity** ^#^	
Hispanic	34 (17.71)
Non-Hispanic	158 (82.29)
**Education**	
< High school	34 (17.71)
High school	43 (22.40)
Some college	57 (29.69)
College or more	58 (30.21)
**Body mass index**	
Normal	21 (10.94)
Overweight	46 (23.96)
Obese	125 (65.10)
**Hypertension medicine**	
Yes	142 (73.96)
No	50 (26.04)
**Smoker**	
Yes	43 (22.40)
No	149 (77.60)
**Cardiovascular disease**	
Yes	44 (24.72)
No	134 (75.28)
**Diabetes**	
Yes	58 (31.35)
No	127 (68.65)

^#^Race and Ethnicity in this study are conceptualized as separate non-mutually exclusive characteristics. Race is operationalized as White or Black and Ethnicity is operationalized as Hispanic or non-Hispanic. For example, an individual can be both Black and Hispanic or Black and non-Hispanic. Race and Ethnicity data is self-reported by participants.

### Results assessment of changes in participant knowledge and behaviors

As depicted in Tables 2, 3, survey results indicate that among participants in the SMBP program there was a high baseline level of knowledge and skills regarding blood pressure management. Despite the high proportion of participants at baseline who answered knowledge questions correctly and indicated that they agreed or strongly agreed with the statements about their hypertension management abilities, post-test results indicated improvement across board on the measures captured in the surveys. Of note, are the increases in self-reported knowledge on how to take blood pressure (pre-test 83% to post-test 99%), understanding how to read blood pressure measurements (pre-test 85% to post-test 100%), and taking their blood pressure medication (pre-test 76% to post-test 86%).

**Table 2:** Participant pre-test/post-test hypertension knowledge.

**Questions**	**Pre-test correct**	**Post-test correct**
	***n*** **(%)**	***n*** **(%)**
Question 1. Which of the following are risk factors for heart disease and stroke? a. Smoking b. High levels of blood cholesterol c. Diabetes d. High blood pressure e. All of the above	204 (88)	179 (91)
Question 2. What are some things you should NOT do when checking your blood pressure? a. Cross your legs b. Take a deep breath c. Have a conversation d. All of the above	203 (84)	175 (89)
Question 3. Which of the following is a normal blood pressure reading? a. 170/80 b. 160/90 c. 120/80	210 (91)	191 (98)
Question 4. What is the top number of a blood pressure reading called? a. Systolic b. Metabolic c. Diabolic	191 (88)	175 (93)
Question 5. What is the bottom number of a blood pressure reading called? a. Diastolic b. Metabolic c. Anabolic	179 (82)	174 (92)

**Table 3:** Pre-test/post-test confidence in hypertension management skills.

**Statement**	**Pre-test**	**Post-test**
	**Agree or strongly agree**	**Agree or strongly agree**
	***n* (%)**	***n* (%)**
Statement 1.	191 (83)	193 (99)
I know how to take my blood pressure. a. Strongly agree b. Agree c. Disagree d. Strongly disagree		
Statement 2.	199 (85)	194 (100)
I understand how to read my blood pressure. e. Strongly agree f. Agree g. Disagree h. Strongly disagree		
Statement 3.	186 (76)	166 (86)
I take my blood pressure medication. i. Strongly agree j. Agree k. Disagree l. Strongly disagree		

### Results assessment of changes in systolic and diastolic blood pressure

Results of our paired t-test analyses of change in blood pressure, presented in Table 4, indicate that, for the overall study population mean systolic blood pressure dropped by 4.48 mm/Hg from the beginning through the end of the study (*p* < 0.05). The association between the intervention and a statistically significant drop in mean systolic blood pressure was found for both women and men as well as for all racial and ethnic groups in the study population. Similarly, significant reductions in mean systolic blood pressure were observed for both those currently on blood pressure medications and those not taking medications. Individuals who had not earned college degrees experienced a significant reduction in mean systolic pressure, while those with college degrees experienced a non-significant decline. Individuals who were overweight or obese experienced significantly lower mean systolic blood pressure, but reductions for participants of normal weight did not reach statistical significance. Program participants who smoked or had diabetes experienced significant decreases in systolic pressure, but those with cardiovascular disease did not.

**Table 4:** Change in mean systolic and diastolic blood pressure mm/Hgb time 1 to time 12.

**Characteristic**	**Mean time 1**	**Mean time 12**	**Mean time 1**	**Mean time 12**
	**Systolic blood pressure**	**Systolic blood pressure**	**Diastolic blood pressure**	**Diastolic blood pressure**
	**mm/Hg**	**mm/Hg**	**mm/Hg**	**mm/Hg**
	**(Std. err.)**	**(Std. err.)**	**(Std. err.)**	**(Std. err.)**
**Overall (*****n*** **=** **192)**	135.36 (1.23)	130.88 (1.19)^*^	82.05 (0.82)	79.32 (0.76)^*^
**Age in years**				
20–39	125.90 (3.14)	119.92 (2.89)	85.23 (3.12)	80.53 (2.00)
40–59	134.22 (2.34)	131.54 (2.37)	84.93 (1.39)	82.41 (1.45)^*^
60–79	136.91 (1.55)	131.79 (1.49)^*^	80.16 (1.04)	77.60 (0.97)^*^
>79	143.68 (7.76)	137.23 (5.61)	78.34 (5.32)	76.05 (3.68)
**Sex**				
Female	134.08 (2.25)	129.53 (1.32)^*^	80.73 (0.92)	78.49 (0.83)^*^
Male	139.56 (1.44)	135.30 (2.58)^*^	86.37 (1.64)	82.05 (1.71)^*^
**Race** ^#^				
Black	136.05 (2.28)	136.05 (2.28)^*^	82.17 (1.85)	79.62 (1.59)
White	135.11 (1.46)	135.11 (1.46)^*^	82.01 (0.89)	79.21 (0.85)^*^
**Ethnicity** ^#^				
Hispanic	131.94 (3.92)	126.23 (3.19)^*^	79.90 (2.20)	77.44 (2.11)
Non-Hispanic	136.10 (1.23)	131.89 (1.26)^*^	82.52 (0.87)	79.73 (0.80)^*^
**Education**				
< High school	130.91 (3.03)	127.16 (3.09)^*^	78.30 (2.09)	76.05 (2.15)
High school	142.28 (3.09)	136.40 (3.09)^*^	84.91 (1.88)	82.44 (1.80)
Some college	136.53 (2.07)	130.29 (1.82)^*^	83.92 (1.18)	79.85 (1.07)^*^
College or more	131.70 (1.80)	129.57 (1.85)	80.30 (1.51)	78.41 (1.28)
**Body mass index**				
Normal	132.35 (3.99)	129.41 (3.23)	81.24 (2.34)	80.44 (1.90)
Overweight	133.31 (2.67)	129.39 (2.58)^*^	81.43 (1.84)	78.97 (1.65)^*^
Obese	136.63 (1.47)	131.68 (1.47)^*^	82.42 (0.99)	79.26 (0.94)^*^
**Hypertension medicine**				
Yes	136.94 (1.44)	132.37 (1.40)^*^	81.81 (0.95)	79.17 (0.91)^*^
No	130.88 (2.28)	126.66 (2.14)^*^	82.74 (1.61)	79.76 (1.35)
**Smoker**				
Yes	142.21 (2.48)	135.76 (2.81)^*^	84.96 (1.92)	80.15 (1.66)^*^
No	133.39 (1.38)	129.48 (1.28)	81.22 (0.89)	79.09 (0.85)^*^
**Cardiovascular disease**				
Yes	140.40 (2.59)	137.10 (2.93)	80.55 (1.63)	78.32 (1.75)
No	132.14 (1.29)	127.48 (1.16)	81.69 (0.93)	78.87 (0.83)
**Diabetes**				
Yes	138.51 (2.26)	133.71 (2.15)^*^	80.88 (1.39)	78.86 (1.47)^*^
No	133.68 (1.52)	129.60 (1.46)	82.82 (1.03)	79.87 (0.90)^*^

* Denotes a statistically significant change in mean systolic or diastolic blood pressure at the p < 0.05 level based on paired t test.

# Race and Ethnicity in this study are conceptualized as separate non-mutually exclusive characteristics. Race is operationalized as White or Black and Ethnicity is operationalized as Hispanic or non-Hispanic. For example, an individual can be both Black and Hispanic or Black and non-Hispanic. Race and Ethnicity data is self-reported by participants.

The results for diastolic blood pressure, presented in Table 4, showed significant reductions, on average 2.73 mm/Hg for the study population over the study period. Significantly lower readings were observed at time point 12 for both males and females and for Non-Hispanics and Whites. Blacks and Hispanics experienced lower mean diastolic blood pressure but did not meet the threshold for statistical significance. Participants between the ages of 40 and 79 experienced statistically significant lower diastolic blood pressure, while the lower readings in blood pressure obtained among the older and younger participants did not reach significance. While lower mean diastolic blood pressures were achieved across education groups, these readings reached significance only for the individuals with some college education. As was seen with systolic blood pressure, diastolic blood pressure was significantly lower in week 12 among overweight and obese individuals but failed to reach significance for participants of normal weight. Both smokers and non-smokers experienced statistically significant drops in diastolic blood pressure as did those taking antihypertensive medications. Statistically significant improvements in diastolic blood pressure were observed for both diabetics and non-diabetics. No significant reductions in diastolic blood pressure were observed for individuals with cardiovascular disease.

### Results assessment of participant experience

Findings from the participant experience surveys, presented in Table 5, illustrate that the SMBP program was both easy to understand and useful in helping participants to increase their knowledge of hypertension and hypertension management. Almost all (99%) either agreed or strongly agreed that the SMBP program was easy to understand. An overwhelming majority indicated that the program helped them to understand facts about hypertension (99% agreed or strongly agreed) and understand the complications of hypertension (99% agreed or strongly agreed). Similarly, most respondents felt that their questions about hypertension were answered and that they know how to use their blood pressure cuff.

**Table 5:** Participant experience survey data (*n* = 189).

**Questions**	**Strongly agree (=4)**	**Agree (=3)**	**Disagree (=2)**	**Strongly disagree (=1)**	**Median (interquartile range)**
	***n*** **(%)**	***n*** **(%)**	***n*** **(%)**	***n*** **(%)**	
Question 1. The workshop helped me to understand facts about hypertension	153 (80.95)	35 (18.52)	0 (0)	1 (0.53)	4 (4, 4)
Question 2. The workshop helped me understand complications of hypertension	153 (80.95)	35 (18.52)	0 (0)	1 (0.53)	4 (4, 4)
Question 3. The workshop did not answer all my questions about hypertension	16 (8.47)	13 (6.88)	61 (32.28)	95 (50.26)	1 (2, 1)
Question 4. The information provided in the workshop was easy to understand	156 (82.54)	32 (16.93)	0 (0)	1 (0.53)	4 (4, 4)
Question 5. I am not able to adequately use my blood pressure cuff at home	9 (4.76)	6 (3.17)	43 (22.75)	131 (69.31)	1 (2, 1)

In addition to completing the participant experience survey, forty-eight percent (48%) of participants completing the SMBP program provided written feedback at the end of the 12-week program. These free form comments provide a qualitative perspective on the program. Three percent (3%) of participants who provided feedback felt the program should include additional visual aids, experienced issues with virtual connectivity, or commented on the time of day a specific workshop occurred. Twenty-two percent (22%) of participants felt the content presented by CHWs was helpful in creating healthier lifestyle habits.

## Discussion

This study evaluates a CHW-led hypertension self-management program implemented among 197 participants in rural northeast Texas. We used three different data sources to evaluate the program. These were: a pre-test knowledge and skills assessment survey, an assessment of change in systolic and diastolic blood pressure before and after the 12-week program, and an assessment of participants' experience with the program after the completion of the intervention. The study found that knowledge about hypertension and hypertension management has improved. Participants showed behavioral change in hypertension management as measured by monitoring their blood pressure at home regularly and taking their medication as prescribed. Changes in both systolic and diastolic blood pressure were observed between mean baseline and last (at the end of the 12-week program) BP measures across subpopulation groups. Finally, participants expressed positive experiences of the program in terms of the information that they were able to obtain as well as the ease of understanding the content.

Knowledge about hypertension and hypertension management improved from baseline measurements. From a conceptual perspective, the increase in participant awareness of the risks of hypertension and understanding of the management of their condition are the first steps toward improving health outcomes. In our study, participants demonstrated increased awareness (from 88% to 91%) that high blood pressure was a risk factor for heart disease and stroke. Similarly, knowledge of proper technique for taking blood pressure (83% to 99%) and interpreting its measurement (85% to 100%) improved from baseline to the week 12 assessment. These findings are conceptually consistent with those found by Boulware et al. (8) where CHW led training was linked to improved hypertension problem solving capabilities including self-management knowledge.

Our findings showed that participants reported an improved behavior to monitor their hypertension and take their medications. The majority (92%) of the participants reported that they were able to adequately use their BP cuffs at home to monitor their blood pressure regularly. Adherence to hypertension medication also improved as 86% of the participants self-reported taking their medications properly after the program compared to (76%) of those who did so before the intervention. These findings are similar to other CHW-led interventions designed to improve disease control and medication adherence. These includes interventions for diabetes and/or hypertension program in Mexico (28), Diabetes Self-Management Education Program in the US (29), and CHW-led intervention in Tanzania in HIV (Human Immunodeficiency Virus) infected pregnant mothers to improve adherence and retention to care (30). The reported behavioral change in most of these programs could be due to CHW's ability to spend more time to educate patients than providers would be able to. Thus, patients are more likely to ask all their questions and be willing to apply the suggested behavioral changes. It is, however, important to examine if these changes are long-lasting past the intervention's completion as most of these studies, including ours, assessed change shortly after the completion of the program. It will be important to continue to monitor graduates from the SMBP program to assess long-term patient adherence as longer monitoring, even at a distance has been reported to increase the likelihood of patient adherence in maintaining a healthy BP range (31). Thus, there is a need for high-quality, rigorous studies to determine long term effectiveness of CHW-led interventions on medication adherence and control of chronic conditions.

Our study participants experienced changes in blood pressure measures (both systolic and diastolic) after the 12-week intervention. Analyses of changes in blood pressure indicated an association between the Self-Management Blood Pressure (SMBP) hypertension program and reductions in both mean systolic and diastolic blood pressure among a sample of 192 individuals from a region of Texas that faces complex, well-documented health challenges (2). The mean difference in baseline and final (at the end of the 12-week program) systolic measure was 4.8 mm/Hg, and the number of diastolic BP was 2.73 mm/Hg. This change was experienced by all patients across all participants. Significant reductions were associated with the program among both men and women and across racial and ethnic groups. While these findings must be viewed cautiously because of design elements, including a relatively small sample size, the lack of a control group, and the reliance on only one pre- and one post-test time points, they do support growing consensus in the literature that CHW-led interventions lighten the community burden caused by chronic diseases (9). Among the most encouraging findings regarding this CHW-led SMBP intervention is the broad-based impact that it is associated with on systolic blood pressure. This is important, as many see systolic blood pressure as the more pivotal marker of hypertension control among older adults such as comprise the overwhelming majority of our study population (32–34). While we did not find significant drops in diastolic blood pressure among people who are over 71 years, the program still has good reach, with all the population strata captured in these data sets showing at least somewhat lower diastolic blood pressure.

These results show that the program had a positive effect on improving certain dimensions of access for rural East Texas community members to information and support to improve their health outcomes. Through the dissemination of information and instruction on a schedule that was flexible and accessible through a distance learning management system, participants were able to self-pace how the information was consumed within the limitations of week-long modules of content. The dissemination of information *via* CHWs who delivered instruction using language, practical examples and vocabulary that were regionally and culturally sensitive to this specific audience. This observation aligns with similar studies that used CHWs to address health outcomes (diabetes and hypertension management) for specific minority populations (35). Regional and cultural sensitivity matters as instructional methods can be tailored to meet the participants on their intellectual, cultural, and social levels.

This study's findings regarding the effectiveness of CHW-led SMBP interventions in combating hypertension in largely rural East Texas are encouraging. They also indicate the need for further research in this field. Introducing a matched control group would enhance the design, as would the incorporation of multiple pre- and post-test measurements of participant blood pressure. Having additional post-test measures would also make it possible to examine the sustainability of the program's influences on blood pressure levels. A final area of promise for future research is in implementation science. A robust examination of the delivery process from the participants and program staff perspectives could reveal areas of strength in the program and areas that need modification.

This study evaluated the efficacy of a SMBP program among rural community participants. Rural communities exhibit disproportionately poorer health outcomes as compared to their urban counterparts (36). Unfortunately, due to the sampling method, racial and ethnic diversity among participants were not under the control of the researchers. This study only evaluated outcomes based on participant self-reported surveys. It may be helpful to use clinical data to confirm changes in blood pressure as measured in a controlled clinical environment. Additionally, participants were identified and recruited *via* convenience sampling methods. Therefore, participants who agreed to join the programs were particularly motivated to self-enroll into the program in order to address their BP conditions. Hence, if we were to align participants' motivations to change their BP-related health behaviors using the Health Behavior Theoretical Model (37)—the participants in our program would be more likely to be in the “cue to action” phase than potential community members who may have been randomly selected to enroll in the program. Participants' readiness to change their behaviors might disproportionately affect the outcomes for the SMPB program. Finally, since this study was based on one group pre-test and post-test analysis, it is not possible to certain that all the changes that were observed were due to the SMBP program. Further studies using rigorous methods are warranted to expand the effects of CHW-led interventions to control cardiovascular diseases and improve outcomes.

## Data availability statement

The raw data supporting the conclusions of this article will be made available by the authors, without undue reservation.

## Ethics statement

The studies involving human participants were reviewed and approved by the University of Texas Health Science Center at Tyler. The patients/participants provided their written informed consent to participate in this study.

## Author contributions

KB developed, supervised, and directed the project. CP was involved with planning, coordinating, and delivery of the project. KB and CP validated the data. PM served as principal investigator of the project. MM completed the statistical analysis of the data. KE and YT provided an analysis of the results and discussion sections. All authors listed have made a substantial, direct, and intellectual contribution to the work and approved it for publication.
